# Fourier Transform Infrared microspectroscopy identifies single cancer cells in blood. A feasibility study towards liquid biopsy

**DOI:** 10.1371/journal.pone.0289824

**Published:** 2023-08-24

**Authors:** Lewis M. Dowling, Paul Roach, Eirik A. Magnussen, Achim Kohler, Srinivas Pillai, Daniel G. van Pittius, Ibraheem Yousef, Josep Sulé-Suso

**Affiliations:** 1 School of Pharmacy and Bioengineering, Guy Hilton Research Centre, Keele University, Stoke-on-Trent, United Kingdom; 2 Department of Chemistry, Loughborough University, Loughborough, Leicestershire, United Kingdom; 3 Faculty of Science and Technology, Norwegian University of Life Sciences, Ås, Norway; 4 Haematology Department, Royal Stoke University Hospital, University Hospitals of North Midlands (UHNM), Stoke-on-Trent, United Kingdom; 5 Histopathology Department, Royal Stoke University Hospital, University Hospitals of North Midlands (UHNM), Stoke-on-Trent, United Kingdom; 6 ALBA Synchrotron Light Source, Cerdanyola del Vallès, Barcelona, Catalonia, Spain; 7 Oncology Department, Royal Stoke University Hospital, University Hospitals of North Midlands (UHNM), Stoke-on-Trent, United Kingdom; The Ohio State University, UNITED STATES

## Abstract

The management of cancer patients has markedly improved with the advent of personalised medicine where treatments are given based on tumour antigen expression amongst other. Within this remit, liquid biopsies will no doubt improve this personalised cancer management. Identifying circulating tumour cells in blood allows a better assessment for tumour screening, staging, response to treatment and follow up. However, methods to identify/capture these circulating tumour cells using cancer cells’ antigen expression or their physical properties are not robust enough. Thus, a methodology that can identify these circulating tumour cells in blood regardless of the type of tumour is highly needed. Fourier Transform Infrared (FTIR) microspectroscopy, which can separate cells based on their biochemical composition, could be such technique. In this feasibility study, we studied lung cancer cells (squamous cell carcinoma and adenocarcinoma) mixed with peripheral blood mononuclear cells (PBMC). The data obtained shows, for the first time, that FTIR microspectroscopy together with Random Forest classifier is able to identify a single lung cancer cell in blood. This separation was easier when the region of the IR spectra containing lipids and the amide A (2700 to 3500 cm^-1^) was used. Furthermore, this work was carried out using glass coverslips as substrates that are widely used in pathology departments. This allows further histopathological cell analysis (staining, immunohistochemistry, …) after FTIR spectra are obtained. Hence, although further work is needed using blood samples from patients with cancer, FTIR microspectroscopy could become another tool to be used in liquid biopsies for the identification of circulating tumour cells, and in the personalised management of cancer.

## Introduction

Liquid biopsy is gaining more interest worldwide as a means to improve the management of patients with cancer. It involves the identification and analysis of circulating tumour DNA, circulating tumour RNA (microRNA, long non-coding RNA, messenger RNA), DNA or RNA from exosomes, and/or circulating tumour cells (CTCs) [1, 2]. The possible applications of liquid biopsy are varied, including early diagnosis and screening of cancer, assessment of treatment response, monitoring patients during follow-up, and even treatment selection through identification of tumours’ subtypes leading to an improved personalised therapy [2].

CTCs, the subject of this work, are cancer cells present in the peripheral blood that originated from the primary tumour or its metastases. These cells can be found as individual cells or as clusters. As individual cells, the number of CTCs in blood could range from 1 to >50 CTCs per 7.5 mL of blood. As clusters, also known as circulating tumour microemboli, they can contain at least 2 CTCs and some of them might contain more than 50 CTCs [3, 4]. Furthermore, together with these CTCs, other cells such as leukocytes, cancer associated fibroblasts, endothelial cells, and platelets can also be present [3, 5, 6]. In the case of lung cancer (the type of tumour studied in this work), different numbers of CTCs’ cut-off in blood have been proposed to link the presence of CTCs in peripheral blood with prognosis and survival [7]. Moreover, the only technique to link the presence of CTCs in blood and cancer prognosis that has gained FDA approval so far is the CellSearch [8].

Difficulty to account for the exact number of CTCs in blood has led to the development of several methodologies to enrich the presence of CTCs in peripheral blood [8]. Broadly speaking, the methods of enrichment can be classified as: 1.) Label-dependent, where CTCs are removed from blood based on the expression of antigens on the surface of CTCs and not by the surrounding blood components (positive CTC selection) or against antigens expressed by blood cells only (negative CTC selection/depletion), and 2.) Label-independent, where CTCs are removed based on their physical properties (cell size, density, deformability, dielectric properties or a combination of physical features) [7, 9]. Methods using positive selection through cancer markers have the disadvantage that those cells not expressing the chosen marker(s) will be lost. In addition, a selection of different antibodies against cancer antigens might be required for different types of cancer, thus, increasing the costs. Making matters more difficult is also the so-called Epithelial–Mesenchymal-Transition (EMT). EMT is a de-differentiation process in which epithelial cells gain mesenchymal traits that confer stem-cell like properties and favour migration [10]. This is associated with the loss of epithelial markers, thus making the isolation of CTCs using antibodies against epithelial markers unsuccessful. All this could also indicate that commonly used methods of positive selection might have been underestimating the numbers of CTCs in blood [11]. Taken together, all this indicates that we are still lacking a robust methodology to identify CTCs in peripheral blood of patients with cancer. Ideally, a method to identify CTCs would require identifying CTCs of most, if not all, tumours, be fast, robust, with high throughput and disturbing to a minimum the management pathway of patients with cancer.

Fourier Transform Infrared (FTIR) microspectroscopy could be a technique that could contain most of these characteristics. It has the ability to identify biochemical changes (at protein, lipid, DNA and RNA level amongst other) in cells and tissues with numerous studies showing that FTIR microspectroscopy can become a diagnostic tool for cancer diagnosis [12]. The application of FTIR microspectroscopy in the identification of CTCs in blood samples would be based on the very different biochemical properties of cancer cells when compared to blood cells. This should, in theory, facilitate the recognition of CTCs in blood. Also, the methodology should disrupt to a minimum the management of patients with cancer. One of the main issues with FTIR microspectroscopy is the substrate where samples are placed. Glass slides of 1 mm thickness used in pathology departments absorb infrared light losing valuable information on proteins, DNA and RNA (the so-called fingerprint region, 1000 to 1800 cm^-1^) with information on lipids remaining (lipid region, 2700 to 3100 cm^-1^) [13–15]. Recently, we developed for the first time a methodology in which the use of thin (0.13–0.17 mm) glass coverslips used in pathology departments as substrates allowed not only the study of the lipid region but also the study of the fingerprint region down to 1350 cm^-1^ [16, 17]. More importantly, we extended this work showing that FTIR microspectroscopy could differentiate between cancer cells and peripheral blood mononuclear cells (PBMC) placed on these glass coverslips [16].

Based on this preliminary work, we carried out the present study in order to assess whether FTIR microspectroscopy could become a tool to be used in the identification and posterior analysis of individual CTCs in peripheral blood. This feasibility study is, to the best of our knowledge, the first time where FTIR microspectroscopy is used to identify individual cancer cells in blood samples placed on glass substrates. Furthermore, it could set up the basis for a protocol to study cancer cells in blood from patients with cancer.

## Materials and methods

### Cells

For this work, the following epithelial cell lines were used: A549 (European Collection of Cell Cultures–ECACC), lung adenocarcinoma, and CALU-1 (ECACC), lung squamous cell carcinoma. Cells were cultured in Dulbecco’s Modified Eagle’s medium (DMEM) with 4.5 g/L glucose and 10% foetal bovine serum (FBS), 5% antibiotic (100x), 5% L-glutamine (200 nM), 1% Hepes buffer solution (1 M), 5% sodium pyruvate (100 nM) and 5% non-essential amino acids (100x) (Merck, UK). Media was changed every three to four days. Cells were passaged by removing culture medium, adding trypsin/EDTA (Merck, UK) and incubating cells for 5 minutes. Following this incubation period, cells were collected and centrifuged at 1200 rpm for 5 minutes. Supernatant was removed and the pelleted cells resuspended in fresh medium. All cells were incubated at 37°C and 5% CO_2_. The standard trypan blue exclusion method was used to assess cell viability. Cells were regularly tested for mycoplasma contamination.

PBMC were obtained through venepuncture from healthy volunteers. The study had ethical approval by the Keele University FMHS Faculty Research Ethics Committee (MH-210190). Healthy volunteers at Keele University participating in this research (older than 18 years of age) gave informed, written consent prior to venepuncture. The volunteers’ data were anonymised linked. Only J.S.-S. had access to these data. Blood samples were collected between 1^st^ December 2021 and 30^th^ November 2022, and the study was carried out between 1^st^ December 2021 and 31^st^ January 2023. Blood (4 mL) was placed in EDTA containing tubes and taken immediately to the laboratory for sample preparation (in the same building).

### Sample preparation

Cancer cells were removed from culture flasks as described above. Following centrifugation at 1200 rpm for 5 minutes, cells were resuspended in 0.9% saline. 10^5^ cancer cells were mixed with 1 mL of blood. In order to remove red cells from blood, 1 mL of blood containing cancer cells was incubated with 10 mL Ammonium-Chloride-Potassium (ACK) lysing buffer (Thermo Fisher Scientific, Loughborough, U. K.) for 5 minutes at room temperature. Blood was then centrifuged at 300 x g for 5 minutes. The supernatant was removed leaving the pellet containing PBMC and cancer cells. The pellet was gently mixed with 5 mL of cold 0.9% saline and centrifuged at 300 x g for 5 minutes. The supernatant was removed, and the pellet resuspended in 0.5 mL 0.9% saline. The resuspended mixed PBMC and cancer cells were immediately used to prepare cytospun samples.

Red cells depleted samples containing PBMC and cancer cells were deposited on glass coverslips (24 x 50 mm, 0.13–0.17mm thickness, GalvOptics, Basildon, U.K.) using a cytospin. Twenty μL of the sample were deposited into the cytospin funnel and the cytospin was ran at 900 rpm for 1 minute. The glass coverslips with the deposited cells were immediately fixed with 100 μL 4% paraformaldehyde (PFA) and incubated for 15 minutes at room temperature. After fixation, samples were washed with 0.9% saline once and deionised water thrice. For each cell line, 3 independent experiments were prepared, and for each independent experiment, 6 samples were set up. Thus, 18 samples were prepared for each cell line. Each independent experiment corresponded to cells at different passage number. For each individual sample 12 to 15 individual cancer cells could be analysed.

### Staining

To confirm the presence of the cancer cells in the doped blood samples a Giemsa stain was used. Giemsa stain is a differential stain containing a mixture of azure blue, methylene blue, and eosin dye. Pathology laboratories commonly use Giemsa staining for blood work such as leukaemia and malaria diagnosis. A staining solution was prepared by diluting a stock Giemsa solution (Atom Scientific) (methanol <25%, glycerol <25%, ethylene glycol <25% and Giemsa powder) at a ratio of 1:40 with a Gurr buffer (Giemsa solution: Gurr buffer) (Thermo Fisher Scientific). The Gurr buffer was produced by adding the Gurr buffer tablet to 100 mL of distilled water as per the manufacturer instructions to produce a pH 6.8 phosphate buffer. The staining solution was dropped onto the sample area of the coverslips and incubated at room temperature for 45 minutes. After this time period, the staining solution was poured off the coverslips and any excess stain was washed off with the Gurr buffer. The samples were air dried to remove excess moisture from the washing. The areas of the samples that were measured with FTIR microspectroscopy were imaged under a microscope to confirm the identity of the cancer cells.

### FTIR microspectroscopy

**Fig 1:**
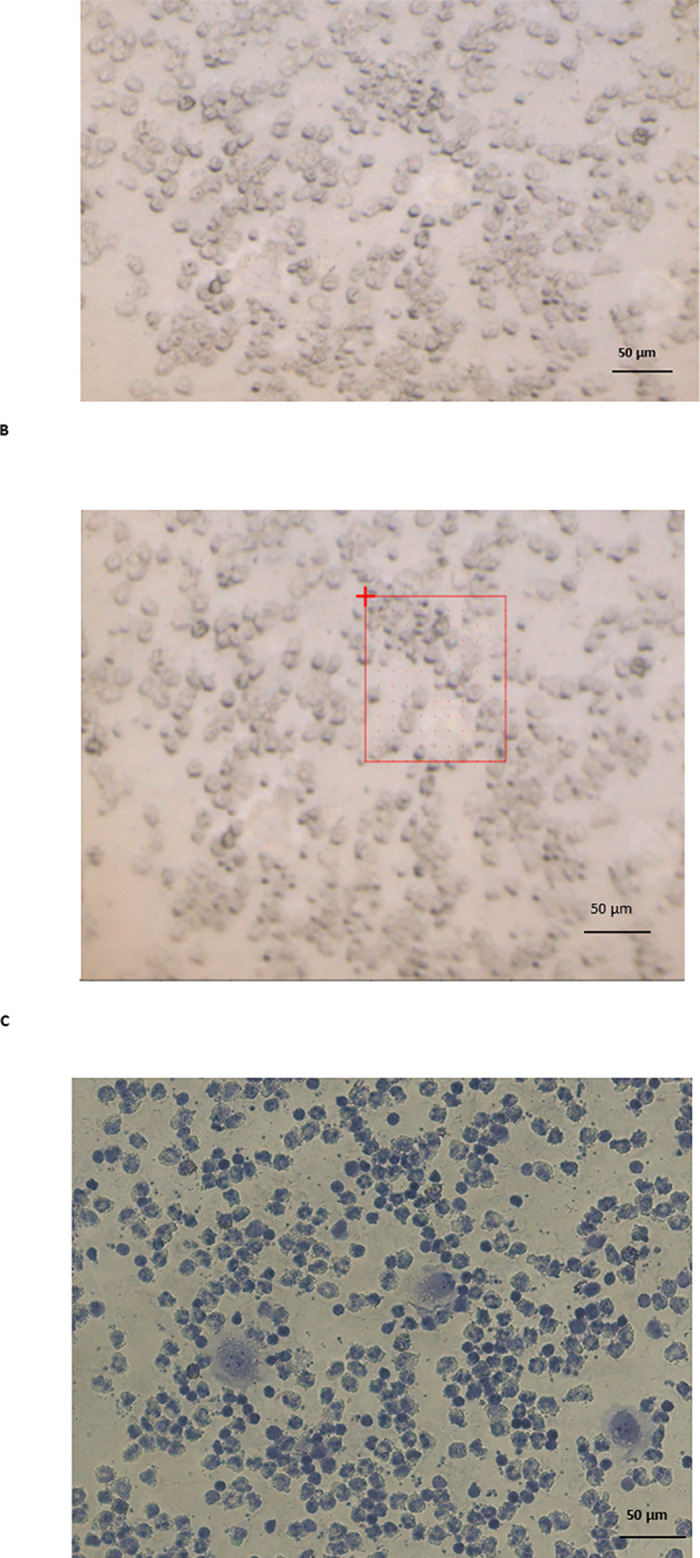
Brightfield image of a cytospun sample containing PBMC and individual A549 cancer cells (A). The same image containing the limits of a map region studied with FTIR spectroscopy (B). The same sample area following staining with Giemsa (C).

IR spectra of the non-stained samples (Fig 1A) were obtained using a Thermo Nicolet iN10(MX) imaging microscope. Developing this methodology into a clinical application will entail mapping areas of blood samples containing individual CTCs. Thus, IR spectra of individual cancer cells were obtained by mapping each individual cancer cell using an aperture size of 15 x 15 μm with a 10 μm stepwise both in the X axis and the Y axis. The size of each individual cancer cell for both cell lines is 20–25 μm diameter. For PBMC, the cell size is 7–10 μm diameter. In order to include the whole individual cancer cell, each map consisted of 16 individual spectra. This method would ensure that at least one 15x 15 μm aperture size spectrum will include only cancer cell and not an area devoid of cell. For PBMC, IR spectra were obtained from clusters of PBMC as individual PBMC did not yield enough IR signal (see discussion). Each map of each PBMC cluster contained 16 IR spectra. The same system was used to map big areas containing both a single cancer cell and groups of PBMC (Fig 1B). These big maps were used to test the methodology and to identify individual cancer cells in blood samples. Spectra were collected at 4 cm^-1^ resolution, with 256 co-added scans. Background measurements were obtained under the same conditions from areas of coverslip without a biological sample. Data pre-processing was carried out as follows: spectra were cropped to the area to be analysed and spectra were processed and corrected for Mie scatter using Extended Multiplicative Signal Correction EMSC as we have previously described [18]. The spectral analysis of cells included the areas between 3500 cm^-1^ and 2700 cm^-1^ for the lipid and amide A (N–H stretching mode of proteins and nucleic acids around 3300 cm^−1^) region, and between 1800 cm^-1^ and 1350 cm^-1^ for the fingerprint region. Data pre-processing was carried out using Quasar software (version 1.7.0)(https://quasar.codes).

### Data analysis

Data analysis was carried out using Quasar software. Principal Component Analysis (PCA) was carried out to identify differences between cancer cells and PBMC. Also, pre-processed data transformed by PCA to reduce dimensions was fed into a Random Forest (RF) classifier. All the single spectrum identified for each individual cancer cell (as described above) and all spectra of PBMC were used as training set. The testing sets were IR maps obtained from areas containing PBMC and one individual cancer cell (Fig 1B shows a representative example). Thus, the method was aimed at assessing whether FTIR microspectroscopy could identify a single cancer cell in blood and surrounded by PBMC.

## Results

The first step in this work was to identify the best cancer cell concentration in blood samples. 10^5^ cancer cells were mixed with 1 mL of blood. The reason to use this cancer cell concentration in blood was to obtain samples with enough number of individual cancer cells to allow data analysis and, at the same time, not to end up with groups of cancer cells clumped together in the final samples as the aim was to study individual cancer cells. Using this methodology, a concentration of 5 x 10^4^ cancer cells per mL of blood yielded very small number of cells to obtain enough data for analysis. On the other hand, 2 x 10^5^ cancer cells per mL of blood yielded groups of cancer cells clustered together and very few individual cancer cells. The clinical application is to use FTIR microspectroscopy to identify single CTCs in blood, thus the methodology was adapted to study single individual cancer cells in blood.

The next step was to obtain the FTIR spectra of both individual cancer cell lines and clusters of PBMC. These spectra were obtained using non-stained samples. However, in spite of the lack of staining, it was easy to identify the individual cancer cells from the PBMC due to its size and shape (Fig 1A). Nevertheless, all samples were stained once the FTIR spectra of individual cancer cells and PBMC clusters were obtained (Fig 1C shows a representative example). Once stained, each individual cancer cell and clusters of PBMC were identified in the stained samples in order to confirm that they corresponded to the same cells studied with FTIR microspectroscopy in the same sample prior to staining. The correlation between the cells studied in the non-stained samples and then checked in the same samples after they had been stained was 100%.

As stated in Materials and Methods, 16 IR spectra were obtained from each individual cancer cell. However, for each individual cancer cell, one spectrum out of the 16 mapping spectra was used for analysis. This spectrum was chosen by identifying the spectrum of each map that contained the highest ratio between amide I and the trough at 1800 cm^-1^. Being the Amide I the most intense absorption band in proteins, the spectrum with the highest ratio would indicate that this spectrum is obtained from an area covering only cell and not areas devoid of cell. Nevertheless, visual analysis was also carried out for each individual cancer cell studied to confirm that the chosen spectrum for each individual cancer cell covered only cell and not areas devoid of cell. For PBMC, all 16 IR spectra of each individual map studying PBMC clusters were used for analysis. Due to the small size of PBMC (7 μm to 10 μm), an aperture of 15 x 15 μm would cover a single PBMC but also an area devoid of PBMC. In this case, it would be easier to separate PBMC from cancer cells rather than when comparing clusters of PBMC and individual cancer cells as we intended here.

**Fig 2:**
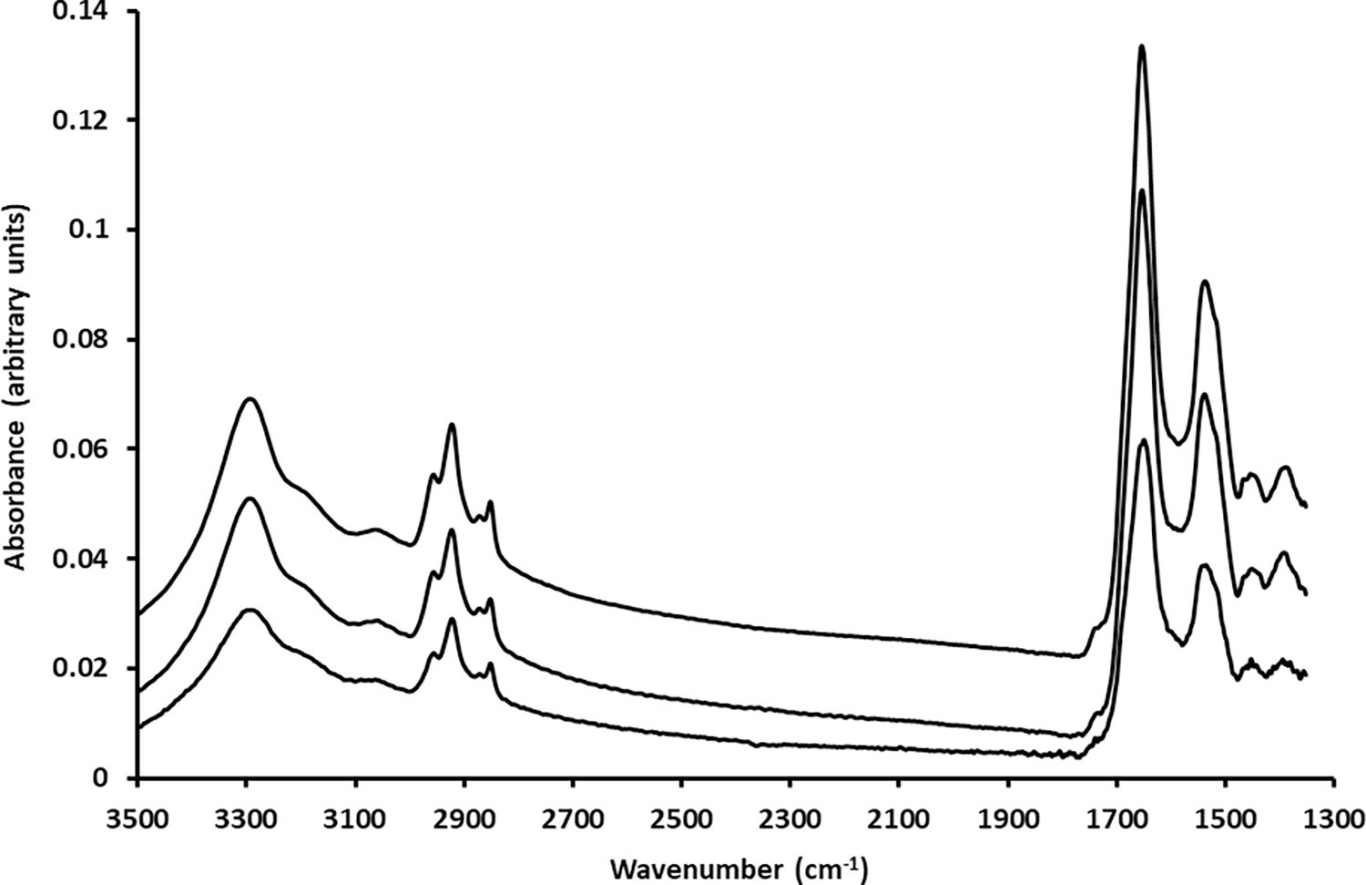
Mean FTIR spectra of A549 cells (235 spectra from 235 individual cells), top spectrum, CALU-1 (265 spectra from 265 individual cells), middle spectrum, and of PBMC (700 spectra), bottom spectrum. Spectra are offset for clarity.

Fig 2 shows the mean spectrum for each lung cancer cell line and PBMC clusters. The number of spectra for PBMC is much higher than for each cancer cell line as the FTIR spectra of the PBMC clusters included those obtained in the samples containing CALU-1 cells and those obtained in the samples containing A549 cells. The main difference between the spectra of cancer cells and the spectrum of PBMC lies in the less intense bands for PBMC for both the lipid and Amide A region and the fingerprint region between 1350 cm^-1^ and 1800 cm^-1^ and the less pronounced shoulder at 1740 cm^-1^ for PBMC when compared to both cancer cell lines.

**Fig 3:**
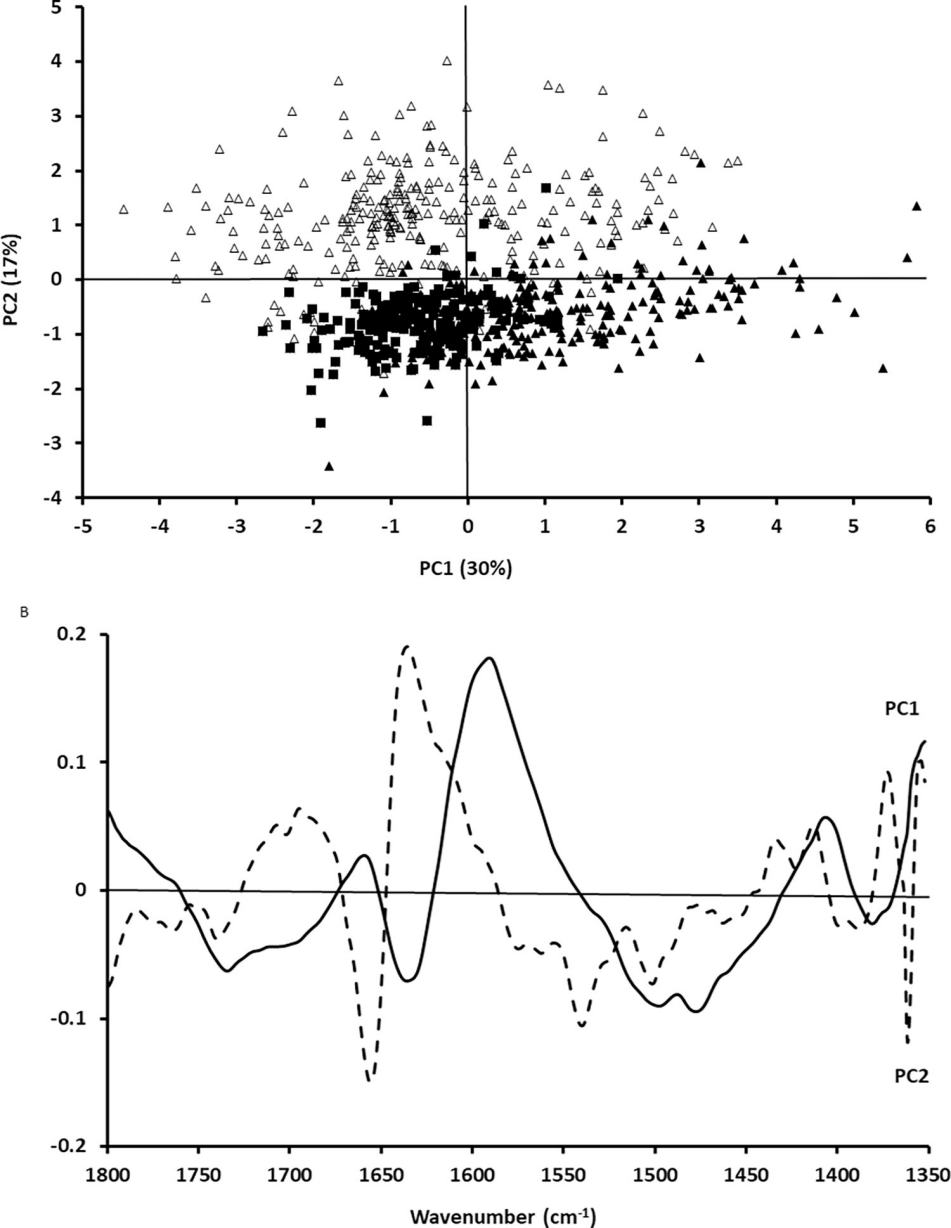
PCA of A549 cells (filled squares), CALU-1 cells (filled triangles) and PBMC (open triangles) for the fingerprint region between 1350 cm^-1^ and 1800 cm^-1^ (A) and corresponding loadings (B).

**Fig 4:**
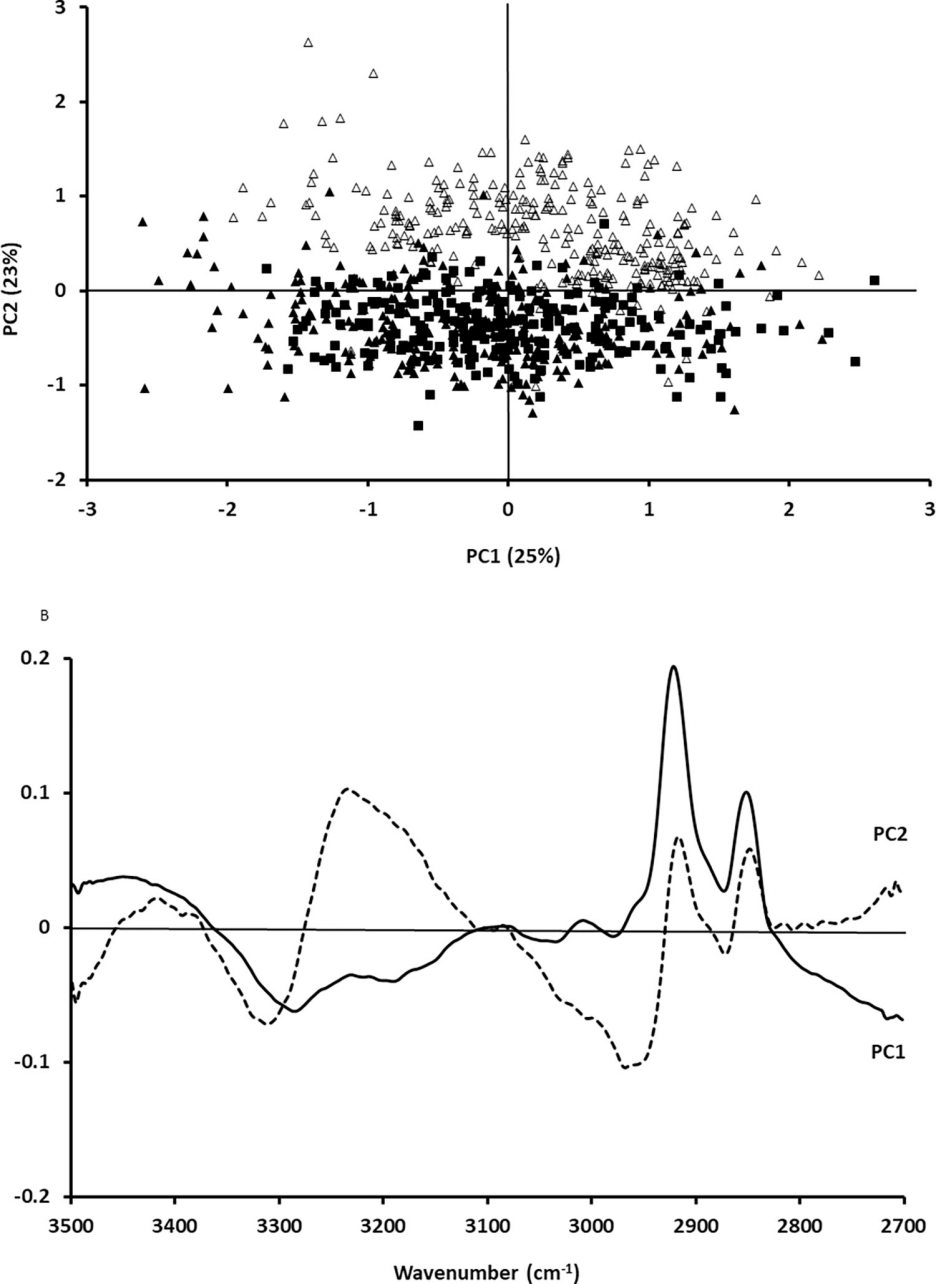
PCA of A549 cells (filled squares), CALU -1 cells (filled triangles) and PBMC (open triangles) for the lipid and Amide A region between 2700 cm^-1^ and 3500 cm^-1^ (A) and corresponding loadings (B).

Once FTIR spectra of cancer cells and PBMC were obtained, the next step was to assess whether PCA could separate cancer cells in the presence of PBMC from these PBMC. Figs 3 and 4 show the PCA and corresponding loadings for the fingerprint region between 1350 cm^-1^ and 1800 cm^-1^ and the lipid and amide A region. As can be seen in Fig 3A, there was a separation between cancer cells and PMBC when the fingerprint region between 1350 cm^-1^ and 1800 cm^-1^ was studied. Similarly, there was also a separation between cancer cells and PMBC when the lipid and Amide A region was analysed (Fig 4A).

**Fig 5:**
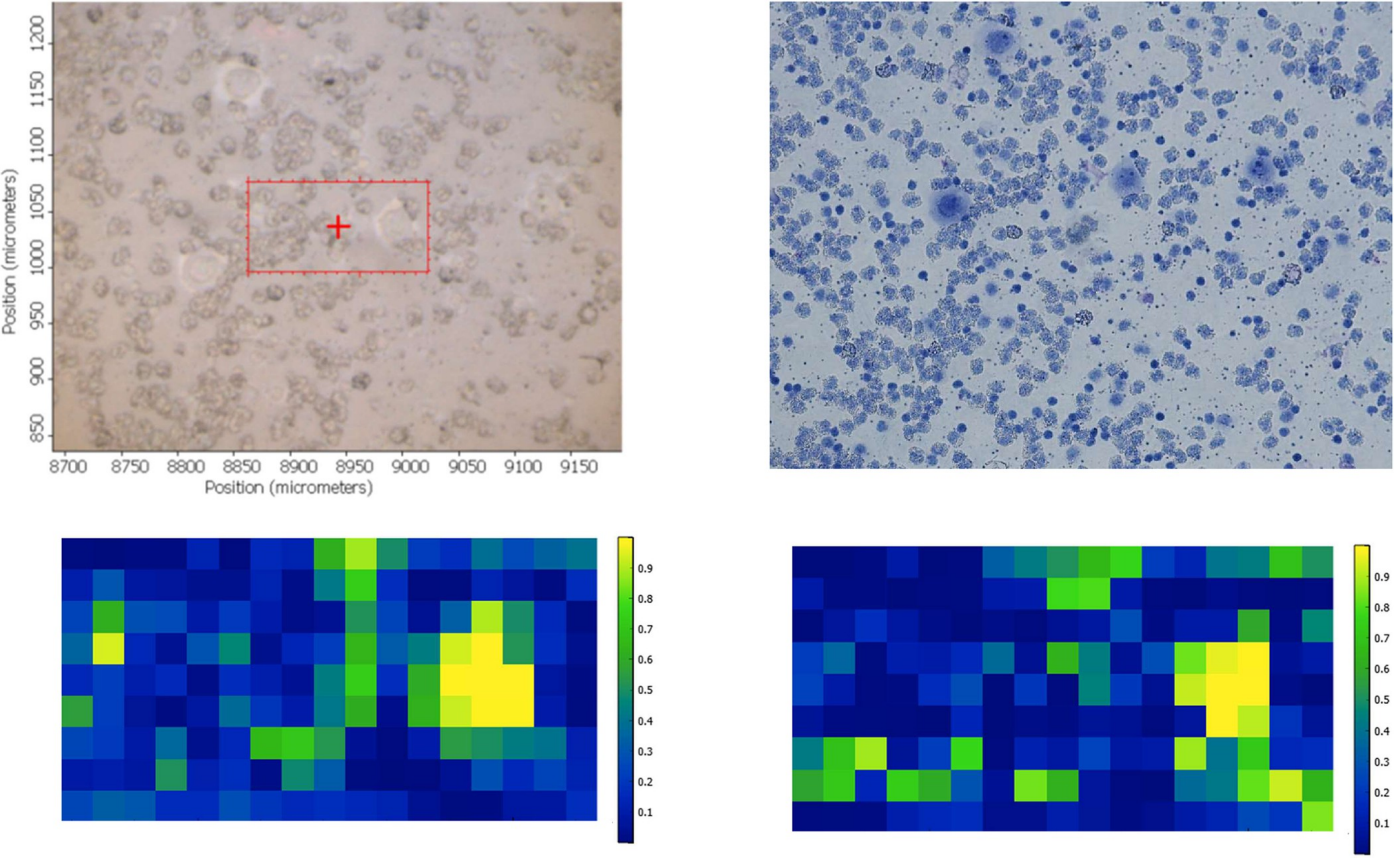
Brightfield image of a cytospun sample containing PBMC and individual CALU-1 cancer cells (A). The same image following Giemsa staining (B). FTIR spectra false colour intensity maps of the spatial rectangular region showed in A for the lipid and Amide A region (C) and the fingerprint region between 1350 cm^-1^ and 1800 cm^-1^ (D).

The application of FTIR microspectroscopy in liquid biopsies will entail the detection of individual cancer cells in blood samples. In order to assess this hypothesis, all FTIR spectra of A549 individual cancer cells and all FTIR spectra of cluster PBMC obtained from the 18 samples mixed with A549 cells were included in the RF training set and used to test for the presence of individual A549 cells in new maps of areas of cytospun blood (devoid of red cells) samples containing PBMC and individual A549 cancer cells. The same procedure was carried out for CALU-1 cell line, i.e., all FTIR spectra of CALU-1 individual cancer cells and all FTIR spectra of cluster PBMC from 18 samples mixed with CALU-1 cells were included in the RF training set and used to test for the presence of individual CALU-1 cells in new maps of areas of cytospun blood (devoid of red cells) samples containing PBMC and individual CALU-1 cancer cells (Fig 1B shows a representative example of such maps). For each cell line, 8 maps to be tested were obtained. We were able to identify a single cancer cell in an FTIR map using either the fingerprint region between 1350 cm^-1^ to 1800 cm^-1^ or the lipid and Amide A region for both A549 and CALU-1 cancer cell lines. Fig 5 shows a representative example. As can be seen in Fig 5C, the individual CALU-1 cell can be identified in the false colour image as presenting with higher intensity when compared to the rest of the studied map.

**Fig 6:**
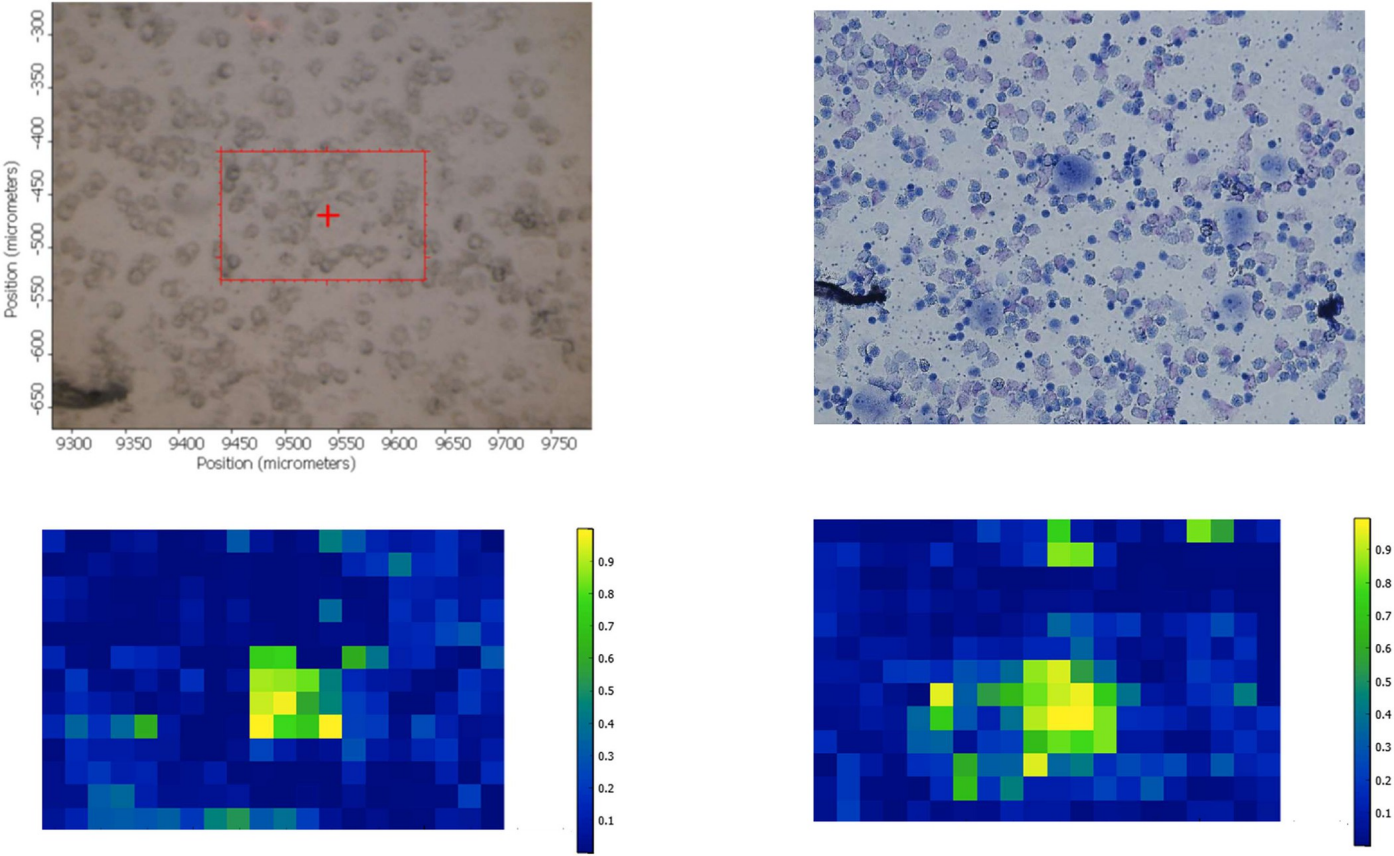
Brightfield image of a cytospun sample containing PBMC and individual CALU-1 cancer cells (A). The same image following Giemsa staining (B). FTIR spectra false colour intensity maps of the spatial rectangular region showed in A for the lipid and Amide A region (C) and the fingerprint region between 1350 cm^-1^ and 1800 cm^-1^ (D).

The next step was to study whether this methodology would be able to identify not only the same type of lung cancer cells used for data training but also cells from a different lung cancer subtype. To this purpose, we used all the spectra of A549 cancer cells as training set to identify individual CALU-1 cells in maps containing PBMC and CALU-1 cells only. Similarly, all the FTIR spectra obtained from CALU-1 cells were used as training set and tested on maps containing PBMC and individual A549 cells only. The data obtained indicated it was possible to use FTIR spectra A549 as training set which was then able to identify single CALU-1 cells. The same applied when FTIR spectra of CALU-1 cells was used as training set in order to identify individual A549 cells. Fig 6 shows a representative example where A549 cells’ FTIR spectra were used as training set to identify a single CALU-1 cancer cell.

## Discussion

Over the last few years, the management of lung cancer has moved rapidly towards a more personalised treatment. The appearance of new immunotherapy and tyrosine kinase inhibitory drugs amongst other, is tailoring the treatment of cancer to each individual patient and tumour [19]. Liquid biopsy, as described above, has the potential to enhance this personalised cancer management. In the case of CTCs, its isolation from blood allows further characterisation of tumours and of potential metastases. However, robust methods to identify and isolate these CTCs in blood are needed.

It is clear that the biochemical properties of CTCs (in their epithelial form or during the EMT process) are very different from the blood cells [10]. It can thus be hypothesised that exploiting these marked biochemical differences should lead to systems that can identify and isolate these CTCs. In the case of FTIR microspectroscopy, most of the *in vitro* work done on its potential clinical application has been aimed at differentiating cancer cells and/or tissues from their non-malignant counterparts. On the other hand, one of its full applications in clinical practice will be the possibility of identifying individual cancer cells in fluids and/or tissues. While this might be complex for tissues which contain different types of cells, and in some cases malignant cells quite similar to non-cancerous cells like in well-differentiated tumours, it would be easier when comparing cancer cells to blood cells.

The study described here shows that when cancer cells are mixed with blood cells, FTIR microspectroscopy can separate between cancer cells and PBMC for both the lipid and Amide A region, and the fingerprint region between 1350 cm^-1^ and 1800 cm^-1^. The lipid region contains the peaks at 2850 cm^−1^ and 2920 cm^−1^ amongst other which correspond mainly to the CH_2_ stretching modes of methylene chains in membrane lipids [20, 21]. On the other hand and within the fingerprint region, the peak at 1740 cm^-1^ corresponds to the C = O stretching mode of phospholipids in cell membranes [22]. It has been described that cancer cells have an abnormal cell membrane composition [23]. In fact, and as can be seen in Fig 2, the shoulder at 1740 cm^-1^ is more intense for both cancer cell lines when compared to PMBC. Furthermore, the metabolism of lipids in cells is different depending on whether these are cancerous or not [24]. Cancer cell division and growth requires a ready and steady supply of lipids to produce cell membranes but also as a source of energy due to its higher proliferation rate when compared to non-malignant cells [25]. This higher presence of lipids in cancer cells (stored in droplets) has been deemed a hallmark of cancer aggressiveness [26]. Thus, it could be argued that lipid content and metabolism in cancer cells could be used to identify cancer cells through the use of FTIR microspectroscopy [27]. Regarding the fingerprint region between 1350 cm^-1^ and 1800 cm^-1^, it contains, amongst other, the Amide I (around 1650 cm^-1^) and amide II (around 1540 cm^-1^) which give information about proteins’ concentration and structure. It is obvious that the protein metabolism of cancer cells from solid tumours differs from that of PBMC. Thus, the data presented here strengthens the view that the use of both the lipid and Amide A region, and the fingerprint region between 1350 cm^-1^ and 1800 cm^-1^ in FTIR microspectroscopy could be good parameters to identify single CTCs in blood. However, the identification of single cancer cells in the big maps was better when the lipid and Amide A region was used as training set when compared to the fingerprint region used as training set. On the other hand, it has to be taken into account that the identification of single cancer cells was easier when the PBMC surrounding cancer cells were scarce rather than present in big clumps. An example is seen in Fig 5A on the left hand side of the rectangular mapping area where a clump of PBMC produces high intensity in the false colour map in Fig 5C. This was the case when the spectral data from the same lung cancer cell line were used for training and testing (for both CALU-1 and A549) but also when CALU-1 cell line spectral data were used as training to identify single A549 cancer cells, and when A549 cell line spectral data were used as training to identify single CALU-1 cancer cells. Taken together, these data indicate that FTIR microspectroscopy could be a tool to identify single cancer cells in blood and that this identification could be made easier when samples have been PBMC depleted. Further work is needed to confirm this using blood from patients with cancer, specially taking into account that these cultured cancer cells will differ from the primary cancer cells present in patients

The next step is bringing this technique to a clinical application. Such development will need for FTIR microspectroscopy to disrupt as little as possible the standard working pathways in pathology departments. The use of glass coverslips for FTIR microspectroscopy as substrates allows further cell characterisation using pathology techniques such as staining (as we have shown in this work, Fig 1C) and/or immunohistochemistry. Coverslips containing cytospins can be glued to standard pathology slides (around 1 mm thickness) and stained using automated systems already available in pathology departments. We tested whether the extra thickness of this sample (standard glass slide plus glued coverslip) would interfere in the automated staining process used in the pathology department at UHNM and this was not the case. Furthermore, once stained (or after immunohistochemistry), the sample can be covered with another coverslip for long storage. The coverslips used in this work can also be exploited to study tissue samples using FTIR microspectroscopy as we have already described [16]. This would allow further studies to assess whether FTIR spectra of lung cancer CTCs are similar to lung cancer cells present in lung tissue from the same patient.

Another issue to be taken into account is the time needed to obtain FTIR spectra and data processing to obtain a result. In this work, maps were obtained to assess the feasibility of this technology to identify individual cancer cells in blood. It took around 15 minutes to obtain the map of a single cell using a 15 x 15 μm aperture with a step size of 10 x 10 μm in X and Y axes. However, in a clinical set up and in order to increase speed, other methodologies such as focal plane array, which covers a bigger area and, hence, is able to obtain FTIR spectra of bigger areas faster, could be used. The methodology could also be modified to increase the speed to obtain a result such as using wider apertures and/or bigger stepwise sizes. Thus, further work is needed to optimise this methodology. Nevertheless, there is no doubt that as this technology develops, faster instruments and faster algorithms to identify these cancer cells in blood can be developed.

In summary, this feasibility study shows it is possible to identify individual lung cancer cells mixed with PBMC using FTIR microspectroscopy. In cases where live CTCs are needed for further characterisation, FTIR microspectroscopy could become a screening tool to identify those blood samples containing CTCs. Further work is now being carried out to assess whether this is the case for other types of tumours, and to improve the algorithms for cancer cell identification in blood.

## References

[1] Connors D, Allen J, Alvarez JD, Boyle J, Cristofanilli M, Hiller C, et al. International liquid biopsy standardization alliance white paper. Crit Rev Oncol Hematol. 2020;156:103112. doi: 10.1016/j.critrevonc.2020.103112 33035734

[2] Aguiar de Freitas AJ, Causin RL, Varuzza MB, Calfa S, Hidalgo CMT, Komoto TT et al. Liquid biopsy as a tool for the diagnosis, treatment, and monitoring of breast cancer. Int J Mol Sci 2022;23(17): 9952. doi: 10.3390/ijms23179952 36077348 PMC9456236

[3] Krebs MG, Metcalf RL, Carter L, Brady G, Blackhall FH, Dive C. Molecular analysis of circulating tumour cells—biology and biomarkers. Nat Rev Clin Oncol. 2014;11(3): 129–144. doi: 10.1038/nrclinonc.2013.253 24445517

[4] Umer M, Vaidyanathan R, Nguyen N, Shiddiky MJ. Circulating tumor microemboli: Progress in molecular understanding and enrichment technologies. Biotechnol Adv. 2018;36(4): 1367–1389. doi: 10.1016/j.biotechadv.2018.05.002 29753882

[5] Bottos A, Hynes NE. Cancer: Staying together on the road to metastasis. Nature. 2014;514(7522): 309–310. doi: 10.1038/514309a 25318518

[6] Ring A, Nguyen-Sträuli BD, Wicki A, Aceto N. Biology, vulnerabilities and clinical applications of circulating tumour cells. Nat Rev Can. 2023;23(2): 95–111. doi: 10.1038/s41568-022-00536-4 36494603 PMC9734934

[7] Syrigos K, Fiste O, Charpidou A, Grapsa D. Circulating tumour cells count as a predictor of survival in lung cancer. Crit Rev Oncol Hematol. 2018;125: 60–68. doi: 10.1016/j.critrevonc.2018.03.004 29650278

[8] Rushton AJ, Nteliopoulos G, Shaw JA, Coombes RC. A review of circulating tumour cell enrichment technologies. Cancers. 2021;3(5): 970. doi: 10.3390/cancers13050970 33652649 PMC7956528

[9] Eslami-S Z, Cortés-Hernández LE, Thomas F, Pantel K, Alix-Panabières C. Functional analysis of circulating tumour cells: the KEY to understand the biology of the metastatic cascade Br J Can. 2022;127(5): 800–810. doi: 10.1038/s41416-022-01819-1 35484215 PMC9427839

[10] Chen L, Bode AM, Dong Z. Circulating Tumour Cells: Moving biological insights into detection. Theranostics. 2017;7(10): 2606–2619. doi: 10.7150/thno.18588 28819450 PMC5558556

[11] Mansilla C, Soria E, Ramírez N. The identification and isolation of CTCs: A biological Rubik’s cube. Crit Rev Oncol Hematol. 2018;126: 129–134. doi: 10.1016/j.critrevonc.2018.03.027 29759554

[12] Baker MJ, Byrne HJ, Chalmers J, Gardner P, Goodacre R., Henderson A. et al. Clinical applications of infrared and Raman spectroscopy: state of play and future challenges. Analyst. 2018;143(8): 1735–1757. doi: 10.1039/c7an01871a 29504623

[13] Bassan P, Mellor J, Shapiro J, Williams KJ, Lisanti MP, Gardner P. Transmission FT-IR chemical imaging on glass substrates: applications in infrared spectral histopathology. Anal Chem. 2014;86(3): 1648–1653. doi: 10.1021/ac403412n Epub 2014 Jan 17. 24410403

[14] Pilling MJ, Henderson A, Shanks JH, Brown MD, Clarke NW, Gardner P. Infrared spectral histopathology using haematoxylin and eosin (H&E) stained glass slides: a major step forward towards clinical translation. Analyst. 2017;142(8): 1258–1268. doi: 10.1039/C6AN02224C 27921102

[15] Rutter AV, Crees J, Wright H, van Pittius DG, Yousef I, Sulé-Suso J. Fourier transform infrared spectra of cells on glass coverslips. A further step in spectral pathology. Analyst. 2018;143(23): 5711–5717. doi: 10.1039/c8an01634h 30351313

[16] Rutter AV, Crees J, Wright H, Raseta M, van Pittius DG, Roach P. et al. Identification of a glass substrate to study cells using Fourier Transform Infrared Spectroscopy: Are we closer to spectral pathology? Appl Spectrosc. 2020;74(2): 178–186. doi: 10.1177/0003702819875828 31517513

[17] Dowling L, Roach P, Rutter AV, Yousef I, Pillai S, Latham D, et al. Optimization of sample preparation using glass slides for spectral pathology. Appl Spectrosc. 2021;75(3): 343–350. doi: 10.1177/0003702820945748 32662291 PMC7961677

[18] Kohler A, Kirschner C, Oust A, Martens H: Extended multiplicative signal correction as a tool for separation and characterization of physical and chemical information in Fourier transform infrared microscopy images of cryo-sections of beef loin. Appl Spectrosc. 2005;59(6): 707–716. doi: 10.1366/0003702054280649 16053536

[19] Krzyżanowska N, Krawczyk P, Wojas-Krawczyk K, Kucharczyk T, and Milanowski J. Immunotherapy in non-small-cell lung cancer patients with driver alterations: A new strategy? Cells. 2022;11(20): 3280. doi: 10.3390/cells11203280 36291146 PMC9600960

[20] Zhou J, Wang Z, Sun S, Liu M, Zhang H. A Rapid method for detecting conformational changes during differentiation and apoptosis of HL60 cells by Fourier-Transform Infrared Spectroscopy. Biotechnol Appl Biochem. 2001;33(2): 127–132. doi: 10.1042/ba20000074 11277866

[21] Lasch P, Boese M, Pacifico A, Diem M. FT-IR Spectroscopic Investigations of Single Cells on the Subcellular Level. Vib. Spectrosc. 2002;28: 147–157.

[22] Holman HN, Martin MC, Blakely EA, Bjornstad K, McKinney WR. IR spectroscopic characteristics of cell cycle and cell death probed by synchrotron radiation based Fourier transform IR spectromicroscopy. Biopolymers. 2000;57(6): 329–335. doi: 10.1002/1097-0282(2000)57:6&lt;329::AID-BIP20&gt;3.0.CO;2-2 11054652

[23] Szlasa W, Zendran I, Zalesińska A, Tarek M, Kulbacka J. Lipid composition of the cancer cell membrane. J Bioenerg Biomembr. 2020;52(5): 321–342. doi: 10.1007/s10863-020-09846-4 32715369 PMC7520422

[24] Butler LM, Perone Y, Dehairs J, Lupien LE, de Laat V, Talebi A et al. Lipids and cancer: Emerging roles in pathogenesis, diagnosis and therapeutic intervention. Adv Drug Deliv Rev. 2020;159: 245–293. doi: 10.1016/j.addr.2020.07.013 32711004 PMC7736102

[25] Ray U, Roy SS. Aberrant lipid metabolism in cancer cells–the role of oncolipid‐activated signaling. FEBS J. 2018;285(3): 432–443. doi: 10.1111/febs.14281 28971574

[26] Beloribi-Djefaflia S, Vasseur S, Guillaumond F. Lipid metabolic reprogramming in cancer cells. Oncogenesis. 2016;5(1): e189. doi: 10.1038/oncsis.2015.49 26807644 PMC4728678

[27] Luanpitpong S, Janan M, Thumanu K, Poohadsuan J, Rodboon N, Klaihmon P. et al. Deciphering the elevated lipid via CD36 in mantle cell lymphoma with bortezomib resistance using synchrotron-based Fourier Transform Infrared Spectroscopy of single cells. Cancers. 2019;11(4): e576. doi: 10.3390/cancers11040576 31022903 PMC6521097

